# Primary fungal iliopsoas abscess caused by *Candida albicans*: a rare clinical entity

**DOI:** 10.1016/j.ijregi.2026.100850

**Published:** 2026-01-29

**Authors:** Hazem Alouani, Ghazi Lâamiri, Manel Yaacoubi, Jasser Rchidi, Mahdi Bouassida, Hassen Touinsi

**Affiliations:** 1 Department of General Surgery, Mohamed Taher Maamouri University Hospital, Nabeul, Tunisia; 2 Faculté de Médecine de Tunis, Université Tunis el Manar, Tunis, Tunisia; 3 Department of Gastro-enterology, Mohamed Taher Maamouri University Hospital, Nabeul, Tunisia

**Keywords:** Iliopsoas abscess, *Candida albicans*, Fungal abscess, Diabetes, Surgical drainage, Groin extension, Fluconazole

## Abstract

**Introduction:**

Fungal iliopsoas abscesses are exceedingly rare and typically occur in immunocompromised individuals. *Candida albicans* is an uncommon pathogen in this location and may lead to delayed diagnosis due to its nonspecific clinical features. Groin extension of such abscesses is even more unusual.

**Case presentation:**

We report a diabetic male patient with a large left iliopsoas abscess extending into the groin. Imaging showed a collection exceeding 10 cm. Initial empirical therapy included fluconazole. Combined surgical and percutaneous drainage was performed. Culture of the drained pus grew *C. albicans*, confirming a fungal iliopsoas abscess. The clinical outcome was favorable.

**Discussion:**

Fungal iliopsoas abscesses represent a rare subset of deep-seated fungal infections. Diabetes is an important predisposing factor. Diagnosis relies on imaging and microbiological confirmation. *C. albicans* is seldom isolated from iliopsoas collections, making this case noteworthy. Optimal management includes drainage and targeted antifungal therapy.

**Conclusion:**

*C. albicans* iliopsoas abscesses are rare and should be suspected in diabetic or non-responding patients. Early identification and tailored antifungal therapy are essential for successful outcomes.

## Highlights

• *Candida albicans* is an extremely rare cause of iliopsoas abscess.
• Diabetes predisposes to hematogenous fungal spread.
• Fungal abscesses may initially mimic pyogenic infections.
• Culture confirmation is essential, especially when empirical antibiotics fail.
• Combined drainage and antifungal therapy lead to excellent outcomes.

## Introduction

Iliopsoas abscess is an infrequent condition that may be primary (hematogenous spread) or secondary to adjacent infection [1]. While bacterial causes—particularly *Staphylococcus aureus*—are most common, fungal iliopsoas abscesses remain exceptionally rare and are typically associated with immunosuppression, prolonged hospitalization, or invasive medical procedures [2].

*Candida albicans* is an uncommon pathogen in this anatomical location, and only isolated cases have been reported. Diabetes mellitus is a recognized risk factor for invasive candidiasis due to impaired neutrophil function and altered host immunity [3].

This report describes a large *C albicans* iliopsoas abscess extending into the groin, highlighting diagnostic challenges and therapeutic considerations. This case has been reported in line with the Surgical Case Report (SCARE criteria) [4].

## Case presentation

A 46-year-old diabetic male with no additional comorbidities presented with a several-week history of progressive left lower abdominal, flank, and groin pain. The pain radiated along the anterior aspect of the thigh and worsened with ambulation and hip flexion. He denied fever, chills, urinary symptoms, or recent gastrointestinal infection. There was no history of trauma, spinal disease, or prior abdominal surgery.

On examination, the patient appeared uncomfortable, maintaining the left hip in slight flexion. There was deep tenderness over the left iliac fossa and along the iliopsoas trajectory, with marked pain during passive hip extension (positive psoas sign). An inguinal swelling was detected, and the patient reported a groin fullness.

Laboratory tests demonstrated moderate inflammatory markers without leukocytosis. Blood glucose levels were elevated but there were no signs of ketoacidosis. Blood cultures were drawn and remained negative.

**Figure 1:**
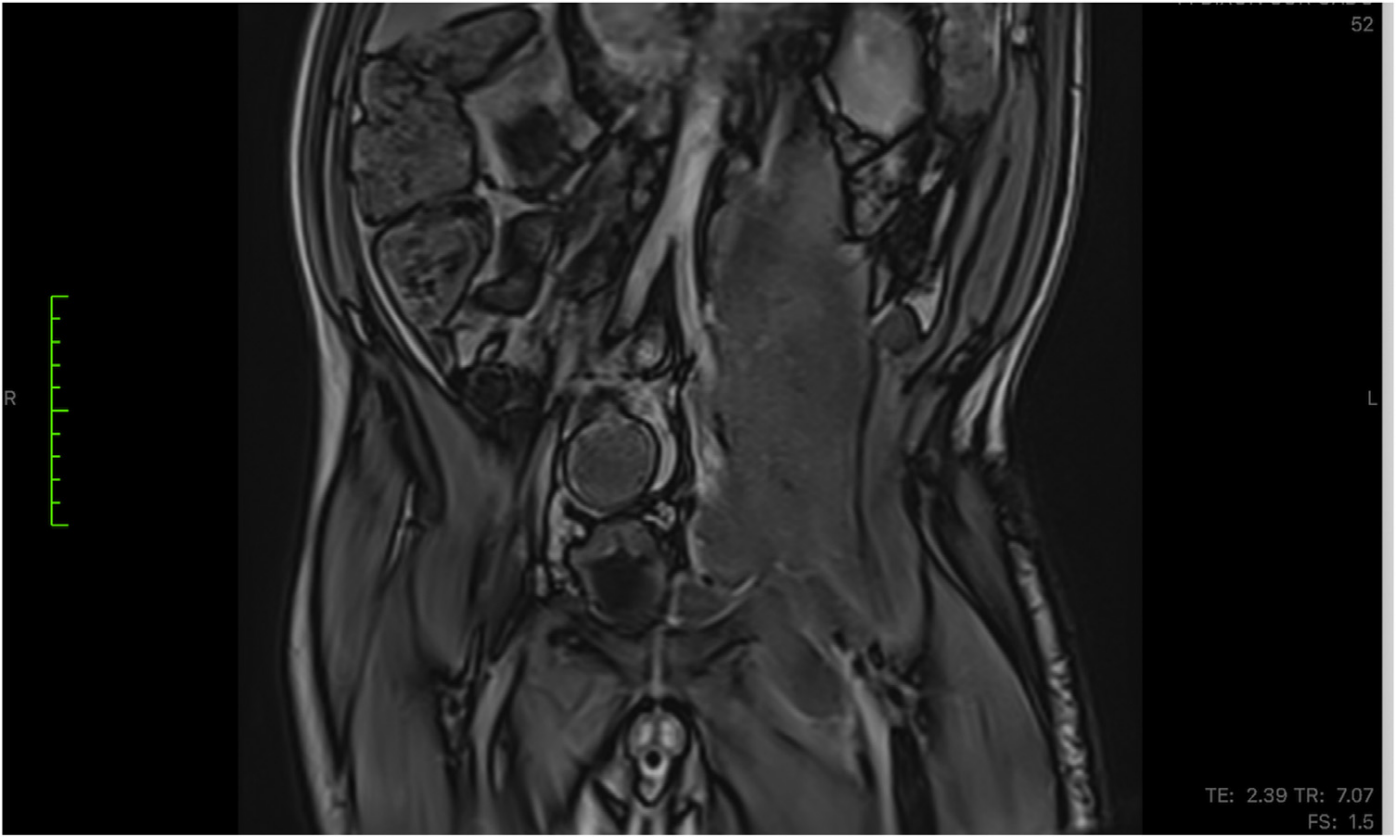
Magnetic resource imaging showing a large left iliopsoas abscess (>10 cm) extending inferiorly into the groin.

An magnetic resonance imaging (MRI) of the abdomen and pelvis revealed a large left iliopsoas abscess exceeding 10 cm, extending inferiorly along the muscle sheath, and reaching the inguinal region (Figure 1). The collection exhibited thickened margins but no gas. No gastrointestinal or vertebral source of infection was identified, suggesting a primary abscess likely related to hematogenous spread.

MRI was selected as the initial imaging modality because it was readily accessible at the time of evaluation and provides superior soft-tissue contrast for defining iliopsoas muscle involvement and extension toward the groin. Given the patient’s stable condition and absence of acute abdominal symptoms, MRI did not delay management and was deemed appropriate for characterizing the collection.

Given the size and inferior extension into the groin, a combined approach was chosen. The patient underwent surgical drainage through an inguinal incision with evacuation of thick purulent material, followed by placement of a wide-bore drain. A computed tomography-guided percutaneous drain was inserted into a deeper loculated portion of the collection. Empirical antifungal therapy with fluconazole was started early due to the atypical presentation and the patient’s diabetic status.

**Figure 2:**
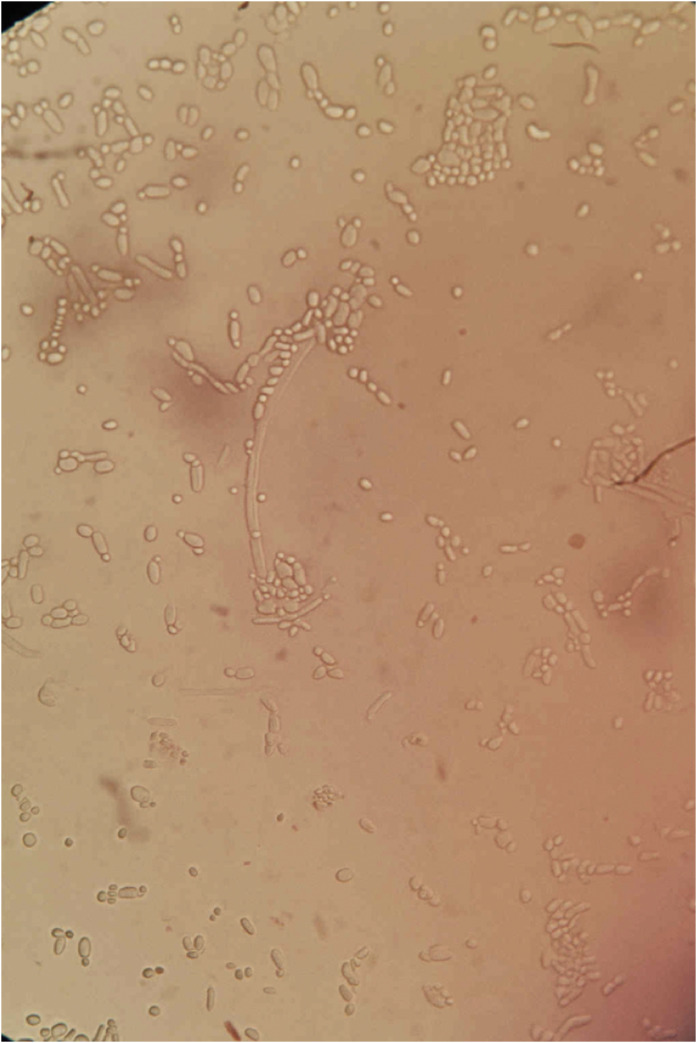
Microscopic appearance showing growth of *Candida albicans* obtained from the drained abscess.

Microbiological analysis of the drained material revealed pure growth of *C. albicans* on Sabouraud dextrose agar (Figure 2). No bacteria were isolated. Blood cultures remained sterile, confirming a primary fungal iliopsoas abscess.

Additional investigations were performed to identify a potential infectious source. HIV serology was negative, and a dental evaluation revealed no oral or odontogenic infection. Colonoscopy was not performed, as the patient had no gastrointestinal symptoms and imaging showed no communication with the bowel or signs suggestive of an underlying colonic pathology.

The patient responded well to drainage and targeted antifungal treatment, with rapid pain improvement and progressive decrease in drain output. Follow-up imaging showed near-complete resolution. The patient received fluconazole at a dose of 400 mg/day intravenously for 7 days, followed by oral fluconazole 400 mg/day to complete a total 4-week course, in accordance with current Infectious Diseases Society of America (IDSA) recommendations for deep-seated candidiasis.

## Discussion

Iliopsoas abscess is a rare but potentially serious condition that may arise from hematogenous spread (primary) or from contiguous infection (secondary) [1]. While *S. aureus* remains the most common pathogen in primary cases, fungal involvement—particularly due to *C. albicans*—is extremely uncommon [2]. The differential microbiological diagnosis of iliopsoas abscesses classically includes *S. aureus* in primary hematogenous cases, and enteric gram-negative bacilli, streptococci, and mixed aerobic–anaerobic flora in secondary abscesses related to gastrointestinal or genitourinary disease. *Mycobacterium tuberculosis* must also be considered, particularly in endemic regions. Fungal etiologies are rare, and most frequently accompany mixed bacterial infections rather than appearing in isolation.

Isolated *Candida iliopsoas* abscesses have been described only sporadically, typically in immunocompromised individuals, intravenous drug users, or patients with prolonged hospitalization [3].

In the present case, diabetes mellitus was the sole identifiable predisposing factor. Diabetes impairs neutrophil chemotaxis, reduces phagocytic function, and facilitates fungal translocation, creating a permissive environment for invasive candidiasis. Hematogenous seeding is the most likely mechanism, especially given the absence of any adjacent infectious source on imaging [5].

Clinical manifestations of iliopsoas abscesses are often nonspecific. The classic triad—fever, back pain, and psoas spasm—is observed in fewer than one-third of patients [6]. Groin extension, as in this case, may mimic inguinal pathology or musculoskeletal disorders, contributing to delays in diagnosis. Pain during hip extension (positive psoas sign) and limitation of hip movement are important clues.

The isolation of pure C. albicans from the drained pus represents strong evidence for a true fungal abscess rather than colonization. The absence of any bacterial growth reinforces the diagnosis of a primary fungal abscess, demonstrating the ability of Candida species to behave as true pathogens in susceptible hosts such as patients with diabetes. Mixed bacterial–fungal infections are more common, and pure fungal abscesses highlight the pathogenic capacity of Candida in susceptible hosts [7].

Optimal management requires drainage—either percutaneous or surgical—combined with systemic antifungal therapy [8]. Large or multiloculated abscesses, especially those extending into the groin, often benefit from a combined approach, as performed in this case. Fluconazole remains the drug of choice for susceptible *C. albicans* strains, offering excellent penetration into soft tissues.

Early recognition of fungal etiology is essential, as empirical antibacterial therapy alone is ineffective and may delay appropriate treatment. With adequate drainage and antifungal therapy, outcomes are generally favorable.

## Conclusion

Large *C. albicans* iliopsoas abscesses extending into the groin are exceptionally rare. Early microbiological identification, prompt drainage, and appropriate antifungal treatment are key to a successful outcome.

## Declaration of competing interest

The authors have no competing interests to declare.
